# Helping family doctors detect vulnerable caregivers after an emergency department visit for an elderly relative: results of a longitudinal study

**DOI:** 10.1186/1471-2296-7-46

**Published:** 2006-07-19

**Authors:** Maida J Sewitch, Mark J Yaffe, Jane McCusker, Antonio Ciampi

**Affiliations:** 1 Department of Clinical Epidemiology and Community Studies, St. Mary's Hospital, and Department of Medicine, McGill University, Montreal, Canada; 2 Department of Family Medicine, St. Mary's Hospital, and Department of Family Medicine, McGill University, Montreal, Canada; 3 Department of Clinical Epidemiology and Community Studies, St. Mary's Hospital, and Department of Epidemiology, Biostatistics, and Occupational Health, McGill University Montreal, Canada; 4 Department of Clinical Epidemiology and Community Studies, St. Mary's Hospital, and Department of Epidemiology, Biostatistics, and Occupational Health, McGill University, Montreal, Canada

## Abstract

**Background:**

Family doctors have been ascribed a role in monitoring patients and their informal caregivers. Little is known about the factors that might alert physicians to changing circumstances or needs of the caregivers. The study objective was to examine changes in family caregivers' quality of life following an emergency department (ED) visit by an older community-dwelling relative that might cue doctors to subsequent caregiver distress.

**Methods:**

A longitudinal study with follow-up at 1- and 4-months was conducted in the EDs of 4 hospitals in Montreal, Canada. Caregivers reported on demographics and quality of life (SF-36). Patients reported on demographics and functional disability. Multiple linear regression for repeated measures was used to evaluate changes in caregiver quality of life and factors related to these changes.

**Results:**

159 caregivers (60.5 yrs ± 15.8%; 73.0% female), including 68 (42.8%) spouses, 60 (37.7%) adult children, and 31 (19.5%) other relatives participated. Following an initial ED visit by older relatives, caregiver general health and physical functioning declined over time, while mental health status improved. Compared to the other relative caregiver group, spouses were at increased risk for decline in general health, mental health, and physical functioning at 1 month, while adult children were at increased risk for decline in physical health at 1 month.

**Conclusion:**

Spouses were most at risk for decline in quality of life. Primary care physicians who become aware of an ED visit by an elderly person may be alerted to possible subsequent deterioration in family caregivers, especially spouses.

## Background

Provision of informal care may be essential for an elderly person to live in the community[1,2] and is associated with reduced home health care use and delayed entry to nursing homes[3]. In the care of seniors, the usual two person (dyadic) doctor-patient encounter frequently expands into a three person (triadic) interaction that also incorporates an informal caregiver, who most often is a family member[4]. In fact, Medalie[5] described family caregivers as "forgotten patients", to reflect the need for a systems approach to both patient and caregiver. He proposed a model of Patient-Caregiver-Physician in which there is ongoing overlap of, and consideration to patient, caregiver, family, doctor, and illness variables[5]. The model suggested that caregiver symptoms, including mood swings, fatigue, headaches, evanescent joint and muscle pains, irritable bowel syndrome, marital and family conflicts, financial problems, depression, etc., may reflect caregiver stress and a need for family doctors to interpret the symptoms and, if appropriate, provide counselling and anticipatory guidance about caregiving[5].

Additional frameworks have been proposed that describe the triadic nature of informal caregiving of the elderly. Kahana et al.[6] suggest a model that includes a spatial axis to represent the "who" (i.e., key individuals involved in caregiving), a transactional axis to describe the "what" (i.e., processes), and a temporal axis to indicate the "when" of caregiving (i.e., length of illness, duration of caregiving, impact of caregiving on developmental and life cycle issues, and the dynamic nature of caregiving). Rolland's Therapeutic Triangle proposed a 3-axis model focussing on illness type, family functioning, and again, time phases[7]. However, since doctors cannot rely on predictability of an illness to understand either what or when a caregiver might be experiencing increasing demands, Rolland's Model evolved into a Therapeutic Quadrangle, in which each illness has its own personality, onset, course, degree of incapacity, predictability, and course[7].

The temporal axis emphasizes that the timing of caregiving can be challenging to an individual's personal life cycle tasks as well as to stages of family life cycle. This has implications for physical, emotional, social, intellectual, and spiritual ways of life of caregivers[5], since caregivers are often elderly and/or ill, or adult children with family and work responsibilities. The longitudinal and evolving nature of the caregiver role led to the identification of caregiving as a "career"[8]. This career may pose risks to caregivers' quality of life [9-13] and mortality[14]. For example, Skaff et al.[15] demonstrated that changes in a caregiver's sense of control or mastery can be affected by whether one is caregiving for a relative at home compared to a facility, or following his or her death. Further, caregivers' responses and behaviours to care recipients with dementia are influenced by when the caregivers began their caregiver careers along the continuum of the disease[16]. Such variability suggests it would be helpful to find ways for primary care doctors to identify subgroups of vulnerable caregivers. Clinicians could become involved with patients or caregivers at different points in an illness life cycle, given that they have broader perspectives of the complete life history of a disease and its constant, relapsing remitting/episodic, or progressive course[7]. Interventions would seem to make a difference, as seen, for example, from a single-blind randomized control trial of a managed program of organized family support services that showed improvement in caregivers' quality of life at 6-months in the treatment versus usual care group[17].

In dealing with varying medical problems in clinical settings, physicians may not respond to caregiver needs [18-20]. Reasons include limitations within health care systems, inter-professional communication problems, and interpersonal conflict amongst members of the health care triad[21,22]. Further, while doctors may relate to the impact that a disease or its restrictions on ADLs have on ill patients, there are few consistent guidelines to help them identify caregivers at increased risk. Lengthy inventories administered on some geriatric assessment units or for research purposes have little practical value for the office-based clinician. Whereas such doctors may benefit from cues such as those provided by the recent findings of Christakis and Allison[23], that hospitalization of an elderly person is a risk factor for death in the spouse, the goal of today's healthcare is to minimize hospital admissions, themselves often preceded by a visit to an emergency department (ED). Following an ED visit the majority of older patients are discharged home[24], many to receive care by family members[25]. Up to 45% of discharged elderly will experience decreased functional autonomy in the months following discharge from the ED [26-28]. Given that most older adults have a primary care physician[24,29] to whom they make an average of 6.3 visits a year[30], these professionals may be well-situated to support vulnerable family caregivers of seniors.

The purpose of this longitudinal pilot study was to examine if the ED visit may signal a turning point for family caregivers' quality of life. Specific objectives were: (1) to describe changes in family caregivers' general health, mental health and physical functioning in the months following their relatives' discharge from the ED, and 2) to determine the caregiver and patient variables related to any changes.

## Methods

This longitudinal pilot study, with follow-up at 1- and 4-months, is a secondary analysis of a multi-center randomized trial in which an ED-based nursing assessment and referral intervention for high-risk elderly discharged home was shown to reduce the risk of subsequent functional decline[31]. The study was conducted at four university-affiliated hospital EDs between September, 1998 and April, 1999. Patients were recruited in the ED by one research assistant, primarily on weekdays during the day shift from among patients aged 65 years and older who were expected to be discharged. Exclusion criteria included the following: referral from a nursing home or chronic care facility, hospital admission expected, inability to communicate in English or French, not a resident of Montreal, medically unstable or cognitively impaired and no family member available to act as proxy, already seen by the hospital's geriatric staff at the same visit, and screening with the Identification of Seniors as Risk (ISAR) indicated a low risk of functional decline in the following six months (see below).

The primary family caregiver for each patient was also invited to participate; non-present caregivers were contacted by telephone. The primary caregiver was identified by the patient as the family member or friend who provided the most assistance with daily activities, regardless of the patient's disability status, and was not paid for the help. For the purpose of the present study, additional inclusion criteria were that each caregiver had to be the same person at all assessments, and baseline and at least one follow-up value of caregiver quality of life had to be available. Excluded from the original caregiver study (N = 193) were 11 (5.6%) patients whose caregivers changed and 23 (11.9%) caregivers who were missing at least one follow-up. Written informed consent was obtained from patients and caregivers. Ethics approval was obtained from participating hospitals prior to study inception.

### Outcomes

The Short Form-36 (SF-36), a 36-item generic measure of quality of life [32,33] in the week prior to the ED visit[34], was used to assess quality of life at baseline, 1- and 4-months. The SF-36 has established psychometric properties and normative data are available that are age and sex standardized for the Canadian population[35]. The measure yields eight subcales including physical functioning, role physical, role emotional, bodily pain, general health perceptions, vitality, social functioning, and mental health. Subscales range from 0 to 100, with higher scores indicating better quality of life. In this study, we focused on three aspects of quality of life: general health, mental health, and physical functioning.

### Independent variables

Caregiver and patient characteristics were assessed by self report at baseline. Caregiver characteristics were: age, sex, relationship to the patient (spouse/child/other relative), and current employment (yes/no). Patient characteristics were: age, sex, education (elementary/other), risk for adverse health outcomes, and functional disability (none to mild/moderate to severe).

### Measures

The *Identification of Seniors At Risk *(ISAR), a 6-item self-report screening tool that was designed to identify elderly persons in the ED at increased risk for adverse health outcomes in the 6 months subsequent to the ED visit[36], was used to screen patients for study inclusion. All patients in this study were at high risk for functional decline (ISAR score of 2 or more out of 6). The *Older American Resources and Services *(OARS) scale is a 14-item assessment of functional disability[37] that was used to report premorbid disability [38]; 7 items assess basic activities of daily living (BADL) and 7 assess instrumental activities of daily living (IADL). Each ADL was dichotomized into completely independent (without use of any assistive device except a cane for walking) and dependent (need for assistance or an assistive device), and disability was summarized as an ordinal scale (0 = none, 1 = mild (IADL dependence only), 2 = moderate (dependent in one to three BADLs), and 3 = severe (dependent in more than three BADLs)).

### Statistical methods

Descriptive statistics were used to characterize caregivers and patients. T-tests were used to compare baseline quality of life to Canadian normative data. Linear regression models for unbalanced repeated measures (mixed model with compound symmetry correlation matrix) were constructed to determine the effect of time on caregiver quality of life. To determine whether there were statistically significant changes in caregiver quality of life over time, we analyzed differences of quality of life variables at 1 and 4 months with respect to the ED visit. At each time point, a multivariate linear regression model was constructed to study the impact on change of caregiver age and patient level of functional disability. All calculations were performed in SAS (Version 9). The procedures PROC MIXED and PROC GLM were used for the multivariate analyses.

## Results

Of the 193 caregivers in the original caregiver study [22], 159 (82.4%) were retained for this research. Participants and non-participants did not differ on age, sex, relationship to the patient, or baseline quality of life.

### Caregivers and patients

Baseline characteristics of caregivers are shown in Table 1. On average, caregivers were 60.5 (standard deviation (sd) = 15.8) years of age and 116 (73.0%) were female. Caregivers included 68 (42.8%) spouses (mean age = 71.9 years), 60 (37.7%) adult children (mean age = 45.4 years), and 31 (19.5%) other relatives (mean age = 64.7 years). Most were born in Canada, married, and not employed outside the home. Median estimated hours of care provided in the previous month were 27.5 for spouses, 11 for adult children, and 10 for other relatives. Table 2 shows that patients had a mean age of 76.6 (sd = 7.4), 85 (53.5%) were female, 50 (31.5%) had an elementary school education, and 69 (43.4%) were moderately to severely functional disabled. The majority had a chief complaint that was of less than 1 week's duration and had been seen in the ED in the previous year. Chief complaints were classified into general symptoms (12.6%), psychiatric/nervous system (10.7%), cardio-respiratory (15.7%), eye, skin, other (5.7%), digestive (18.9%), genito-urinary (5.0%), musculoskeletal (17.0%), diseases (5.7%), injuries (7.6%), and other (1.3%).

**Table 1:** Caregiver Characteristics at Baseline (N = 159)

**Characteristics**	**N (%)**
Age (mean ± sd)	60.5 (15.8)
Female	116 (73.0)
Married	109 (69.0)
Elementary school education	50 (31.4)
Language of interview	
English	70 (45.3)
French	87 (54.7)
Born in Canada	127 (80.9)
Relationship to care receiver	
Spouse	68 (42.8)
Adult child	60 (37.7)
Other relative	31 (19.5)
Work outside home	48 (30.2)

**Table 2:** Patient Characteristics at Baseline (N = 159).

**Characteristics**	**N (%)**
Age (mean ± sd)	76.6 ± 7.4
Female	85 (53.5)
Elementary school education	50 (31.5)
High risk of functional decline*	72 (45.3)
Functional disability**	
None	42 (26.4)
Mild	48 (30.2)
Moderate	52 (32.7)
Severe	17 (10.7)
Chief complaint	
*Symptoms*	
General symptoms	20 (12.6)
Psychiatric/nervous system	17 (10.7)
Cardio-respiratory	25 (15.7)
Eye, skin, other	9 (5.7)
Digestive	30 (18.9)
Genito-urinary	8 (5.0)
Musculoskeletal	27 (17.0)
*Diseases*	9 (5.7)
*Injuries*	12 (7.6)
*Other*	2 (1.3)
	
Duration of chief complaint	
Less than 1 week	118 (74.7)
1 week to 1 month	25 (15.8)
More than 1 month	15 (9.5)
ED visits in previous year (0/1)	122 (76.7)

*assessed with the ISAR

**assessed with the OARS

### Caregiver quality of life

Table 3 presents the mean scores on caregiver quality of life at baseline, 1-month, and 4-months. Across all caregivers and among spousal and adult child caregivers, general health and physical functioning declined over time while mental health improved. For other caregiver relatives, general health improved at 1-month and declined at 4-months, and mental health and physical health improved at both follow-up assessments. Significant effects of time were seen for general health for all caregivers (p = .008) and adult child caregivers (p = .008), and for mental health for all caregivers (p = .009) and spousal caregivers (p = .029).

**Table 3:** Mean (standard deviation) scores over time on quality of life of family caregivers of seniors treated in the emergency department.

**Quality of life measure**	**Baseline** **N = 159**	**1-month** **N = 136**	**4-months** **N = 127**	p-value*
General health	*77.3 *(*20.5*)	*72.2 *(*23.7*)	*72.0 *(*21.5*)	.*008*
Spouse	74.0 (19.8)	70.5 (19.8)	70.4 (22.0)	.237
Adult child	80.9 (19.9)	69.5 (26.4)	72.3 (22.3)	.008
Other relative	77.4 (22.4)	79.7 (20.7)	76.0 (18.7)	.940

Mental health	*72.5 *(*20.3*)	*74.7 *(*19.8*)	*76.6 *(*18.7*)	.*009*
Spouse	70.4 (21.5)	70.6 (21.5)	74.8 (17.9)	.029
Adult child	72.9 (19.2)	75.6 (18.8)	76.7 (20.1)	.308
Other relative	76.5 (19.8)	80.5 (17.0)	81.7 (17.6)	.144

Physical functioning	*80.4 *(*24.4*)	*78.2 *(*24.6*)	*78.4 *(*22.4*)	.*263*
Spouse	75.5 (25.8)	72.9 (26.5)	73.9 (23.1)	.439
Adult child	89.8 (16.9)	80.8 (24.6)	85.3 (19.1)	.261
Other relative	73.1 (28.3)	83.1 (19.7)	76.0 (24.0)	.659

* p-value for mixed models testing the effect of time (with covariance structure: compound symmetry)

Tables 4 and 5 present the results of the linear regression analyses for changes in family caregivers' quality of life at 1- and 4-months. Results are presented for all caregivers (overall) as well as stratified by location of the caregiver interview. At 1-month, compared to other relatives spousal caregivers had statistically significantly poorer general health and physical functioning and marginally poorer mental health, and adult children had statistically significantly poorer physical functioning. Compared to other relatives, average scores in spouses were 9.2 points lower on general health, 10.1 points lower on physical functioning, and 7.1 points lower on mental health, and 13.7 points lower on physical functioning in adult children. Baseline telephone interviews were associated with poorer outcomes compared to interviews conducted in the ED. Compared to other relatives, spouses had statistically significantly poorer mental health and adult children had statistically significantly poorer physical functioning. At both 1- and 4- months, caregivers of patients in the intervention group reported poorer general health that was more pronounced in caregivers interviewed in the ED.

**Table 4:** Results of linear regression models for change in quality of life at 1-month in family caregivers of seniors treated in the emergency department.

**QUALITY OF LIFE MEASURE**	**1 MONTH**					
	**OVERALL**		**ED**		**TELEPHONE**	
	**B**	**p-value**	**B**	**p-value**	**B**	**p-value**
**General Health**						
Caregiver age*	.2	.25	.1	.52	.1	.87
Relation to patient						
Spouse	-9.2	.04	-7.3	.21	-11.2	.15
Adult child	-7.9	.13	-7.7	.25	-14.3	.16
Other relative	1		1		1	
Patient disability						
None – mild	1		1		1	
Moderate – severe	-1.4	.66	-4.6	.28	2.8	.62
Intervention	-6.6	.04	-9.6	.03	-2.2	.68

**Mental health status**						
Caregiver age*	.1	.49	.1	.60	-.1	.75
Relation to patient						
Spouse	-7.1	.08	-1.8	.76	-14.6	.01
Adult child	-1.3	.78	-1.4	.84	-7.3	.33
Other relative	1		1		1	
Patient disability						
None – mild	1		1		1	
Moderate – severe	3.2	.28	1.2	.78	4.9	.25
Intervention	-4.7	.10	-5.3	.24	-3.0	.46

**Physical functioning**						
Caregiver age*	-.2	.38	-.1	.79	-.5	.14
Relation to patient						
Spouse	-10.1	.04	-7.6	.22	-13.8	.11
Adult child	-13.7	.02	-8.1	.25	-27.2	.02
Other relative	1		1		1	
Patient disability						
None – mild	1		1		1	
Moderate – severe	-.18	.96	-4.0	.38	6.0	.34
Intervention	-.7	.84	.2	.96	.9	.87

* increase of 1 year of age

At 4-months, caregivers of patients in the intervention group who were interviewed in the ED also reported poorer mental health compared to caregivers of patients in the usual care group. For caregivers interviewed in the ED, age was marginally related to improved general health at 4-months. Patient disability was unrelated to changes in caregiver quality of life.

## Discussion

Given the importance of family caregivers in the care of the elderly, the main objective of this exploratory study was to determine the relationship between patient and caregiver characteristics and changes in family caregivers' quality of life following an ED visit by an elderly person. The most important finding was that spouses were most at risk for decline in quality of life compared to other relative caregivers. At nearly all assessments, spouses rated poorest on general health, mental health and physical functioning compared to other caregiver subgroups. Because quality of life decreases with increasing age[35], spouses' lower quality of life may be attributed to their: 1) older age compared to other relatives, 2) providing more caregiving time, which was more than double that of other caregiver subgroups, and/or 3) increased emotional investment, due to their proximity and relationship history with the care recipient. Spouses may have been more available to provide assistance but less able to distinguish time spent providing care from time spent with the patient. In addition, if the length of time that people have been married is viewed as an indicator of bonds of reciprocity or strength of interpersonal ties[39], then the effects of caregiving on elderly spouses may be related to spouses' emotional investment, on-going physical proximity, or relationship history with the care recipient.

We also found that adult children were at highest risk for poorer physical functioning at 1-month compared to other caregiving relatives. Collectively, our findings suggest that the closest relatives experienced the greatest decline in quality of life in the months following the ED visit. Moreover, location of the caregiver interview was important. For caregivers interviewed by telephone, spouses and adult children fared worse on mental health and physical functioning at 1-month, respectively, compared to other relatives. Thus, the lack of accompanying an elderly relative to the ED may be attributable to the caregiver's own health problems.

A second study objective was to examine changes in caregiver quality of life following the ED visit. The observed decline (from baseline) at 4-months in general health and physical functioning of spouses and adult children may imply that neither aspect of quality of life returned to the pre-morbid state 4-months post-ED visit, since quality of life was derived from perceptions of the week prior to the ED visit. Notwithstanding that the changes were modest, these findings are noteworthy because maintaining the physical health and functioning of caregivers allows them to continue with their caregiving responsibilities[40].

Although population-based studies suggest that healthy, community-based older adults do not "burden" their caregivers[40], our study implies that during certain transition points such as ED visits, families are potentially at risk for negative outcomes[41]. In the present study, community-dwelling elderly treated in the ED for medical problems that did not entail hospitalization required assistance from family in the months following discharge that may have led to deterioration in their own quality of life. The toll of an uncomplicated ED visit by a frail elderly relative on the caregivers' quality of life may result in a cascade of events including inability of the caregiver to provide further assistance and earlier ED readmission and hospitalization of the patient, since older adults are at risk for early ED readmission[42] and not having an informal caregiver is an independent risk factor for hospitalization in community-dwelling elderly[43]. Primary care physicians may intervene with the caregiver subgroup most at risk for declining quality of life. Often aware of when their elderly patients seek health care in the ED, primary care physicians could suggest that the spouse come in for a check up or to discuss how s/he is handling things at home, and/or point out resources that are available in the community. With the advent of telehealth, additional opportunities for interventions designed to support vulnerable caregivers will become available[44].

The main study limitation is that findings may not be generalizable to all caregivers of elderly relatives treated in the ED as patients were limited to those visiting during the day shift only and deemed well enough to be discharged. Because study patients had medical problems that did not require hospital admission, our findings likely underestimate the true extent of the impact of an ED visit on caregiver quality of life. One strength of this research is the longitudinal design that allowed for the evaluation of changes in caregivers' outcomes, although our observation period of 4 months might be viewed as relatively short in the caregiver careers of some individuals. As a pilot project, caregiver outcomes of the ED visit were not compared as a function of the caregivers' illnesses. Hence future research might include a study of longer duration that contains variables such as care recipients' illnesses and the duration of the caregiver experience.

In conclusion, this study of family caregivers of older adults discharged from hospital ED for mainly acute problems found that caregivers' general health and physical functioning declined and mental health improved in the first 4-months following discharge. Spousal caregivers were most at risk for poorer quality of life compared to other caregiver subgroups. These findings may offer clues to primary care physicians that would alert them to vulnerable caregivers, in particular spousal caregivers. A combination of family stress theory and family systems theory may be applied for caregiving families to use resources and to develop coping capabilities to meet the demands of a caregiving role. Inasmuch as these physicians commonly handle large volumes of patients, our findings may help family physicians establish criteria for prioritizing their time and effort by providing some insight into particular patient subgroups that might benefit from increased attention.

## Abbreviations

ED Emergency department

## Competing interests

The author(s) declare that they have no competing interests.

## Authors' contributions

Dr. Maida Sewitch was responsible for performing the statistical analyses and preparation of the manuscript.

Dr. Mark Yaffe was responsible for preparation of the manuscript.

Dr. Jane McCusker was responsible for the design and conduct of the study.

Dr. Antonio Ciampi was responsible for overseeing the statistical analyses.

All authors contributed to the interpretation of the study findings and approved the final manuscript.

**Table 5:** Results of linear regression models for change in quality of life at 4-months in family caregivers of seniors treated in the emergency department.

**QUALITY OF LIFE MEASURE**	**4-MONTHS**					
	**OVERALL**		**ED**		**TELEPHONE**	
	**B**	**p-value**	**B**	**p-value**	**B**	**p-value**
**General Health**						
Caregiver age*	.2	.17	.4	.08	.1	.75
Relation to patient						
Spouse	-6.7	.17	-8.6	.18	-7.7	.34
Adult child	-3.2	.60	-2.3	.75	-7.0	.56
Other relative	1		1		1	
Patient disability						
None – mild	1		1		1	
Moderate – severe	2.7	.42	5.8	.18	.5	.94
Intervention	-5.8	.08	-8.5	.05	-2.8	.60

**Mental health status**						
Caregiver age*	-.1	.45	-.1	.63	-.1	.63
Relation to patient						
Spouse	-2.4	.57	-1.4	.81	-5.8	.41
Adult child	-6.4	.24	-9.3	.16	-5.0	.63
Other relative	1		1		1	
Patient disability						
None – mild	1		1		1	
Moderate – severe	2.1	.48	3.0	.44	1.6	.76
Intervention	-3.7	.20	-7.3	.06	-.3	.95

**Physical functioning**						
Caregiver age*	.1	.68	.1	.87	.1	.98
Relation to patient						
Spouse	-5.9	.22	-9.2	.15	-.9	.91
Adult child	.2	.98	-1.2	.87	-3.0	.80
Other relative	1		1		1	
Patient disability						
None – mild	1		1		1	
Moderate – severe	-1.6	.61	-5.6	.19	3.9	.50
Intervention	-.8	.82	-.5	.90	-.4	.84

adjusted for caregiver's baseline outcome score;

* increase of 1 year of age

## Pre-publication history

The pre-publication history for this paper can be accessed here:


